# Atypical Presentation of Coronary Artery Fistula: Case Report and Review of the Literature

**DOI:** 10.7759/cureus.7735

**Published:** 2020-04-19

**Authors:** Mohinder Vindhyal, Lohitha Kolli, Naveen Kannekanti, Venkata Boppana

**Affiliations:** 1 Internal Medicine, University of Kansas School of Medicine, Wichita, USA; 2 Radiology, University of Kansas School of Medicine, Wichita, USA; 3 Pediatrics/Neonatal & Perinatal Medicine, MetroHealth Medical Center, Case Western Reserve, Cleveland, USA; 4 Cardiology, University of Kansas School of Medicine, Wichita, USA; 5 Cardiology, Heartland Cardiology, Wichita, USA

**Keywords:** coronary artery fistula, angina

## Abstract

Chest pain is one of the common complaints a patient presents to the healthcare provider. It needs prompt evaluation to determine the cause and origin. Angina occurs when myocardial oxygen demand exceeds oxygen supply; the clinical manifestation is often chest discomfort. Atherosclerotic disease is the major cause of angina. However, several non-atherosclerotic conditions have been studied and reported in the literature that causes angina in rarity. We describe a case of coronary artery fistula (CAF) likely causing angina.

## Introduction

Coronary artery fistulas (CAFs) are one of the significant congenital anomalies bypassing the myocardial capillary bed forming a communication between a coronary artery and either a chamber of the heart or any systemic or pulmonary circulation [1] The incidence of CAF and coronary anomalies is anywhere around 0.002% and 0.2 to 1.2% of the general population [2-3]. Coronary artery anomalies are mainly abnormalities of origin, distribution, and termination [4]. CAF is primarily due to congenital anomalies but can be acquired either due to a disease-related condition, traumatic or iatrogenic [5].

## Case presentation

A 40-year-old Chinese male presented to the emergency department complaining of chest pain. The patient reported that he felt the pain while lifting a weight of 40 pounds in the kitchen. The chest pain was heavy, retrosternal, constant for 30 minutes, non-radiating associated with dizziness, nausea, and palpitations. It relieved gradually with rest after presenting to the emergency room. On further questioning, the patient revealed having similar episodes of chest pain for the past several years, limiting his physical activity (CCS Class II angina) but did not seek medical attention.

**Figure 1:**
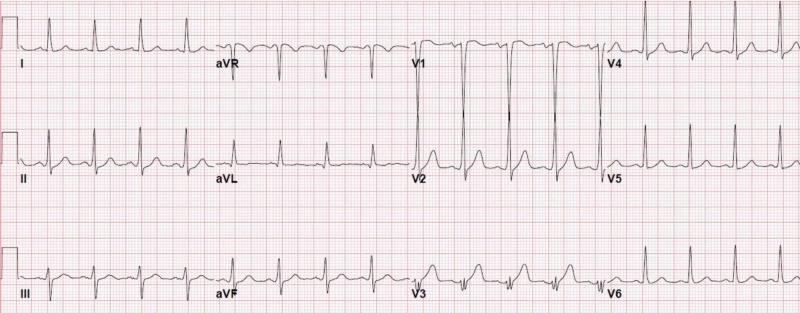
EKG showing sinus tachycardia EKG, electrocardiogram

The patient’s medical history was significant for gastroesophageal reflux disease but was not on medications. His social history revealed he was a worker in a restaurant and had ten pack-years of smoking history without any alcohol or illicit drug use. The patient denied having any significant family history. The patient’s physical exam revealed continuous grade II/VI murmur at left sternal border, otherwise clear lungs, normal jugular venous pressure, and no pedal edema. The patient’s initial troponin was negative, and electrocardiogram (EKG) findings showed mild tachycardia, as seen in Figure 1.

The differential for a patient presenting with chest pain and continuous murmur includes ruptured sinus of Valsalva aneurysm, patent ductus arteriosus, anomalies of coronary artery origin, aortopulmonary septal defect, ventricular septal defect, Lutembacher syndrome, and CAF.

Investigations

The patient underwent a chest X-ray which ruled out pneumothorax and did not show any widening of the mediastinum. Bedside point-of-care ultrasound ruled out any tamponade physiology. The patient's Pulmonary Embolism Rule-out Criteria (PERC) score for pulmonary embolism was zero, and no workup was done. The patient was admitted to the medical floor for further workup. The primary admitting team risk stratified the patient for probable cardiovascular disease. The patient then underwent an exercise stress test as a part of his cardiology workup. The stress test was prematurely terminated at seven minutes as the patient developed dizziness, ST-segment depression in the lateral leads, non-sustained monomorphic ventricular tachycardia and a transient left bundle branch block. Cardiac catheterization showed insignificant coronary artery disease but revealed a fistula leading from the left coronary artery (LCA) near the bifurcation to the main pulmonary artery (PA) as seen in Video 1.

**Video 1:** CAF arising from the left coronary artery to the main pulmonary artery CAF, coronary artery fistula

**Figure 2:**
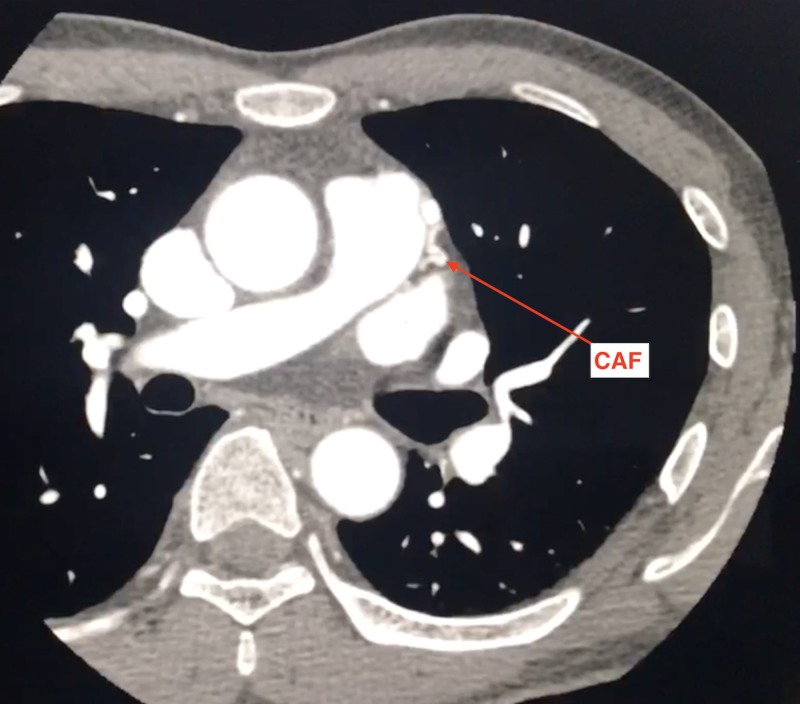
Axial CTA: coronary artery fistula seen as tortuous vessels with aneurysmal dilatation along the left lateral aspect of PA trunk CTA, computed tomography angiography; PA, pulmonary artery

**Figure 3:**
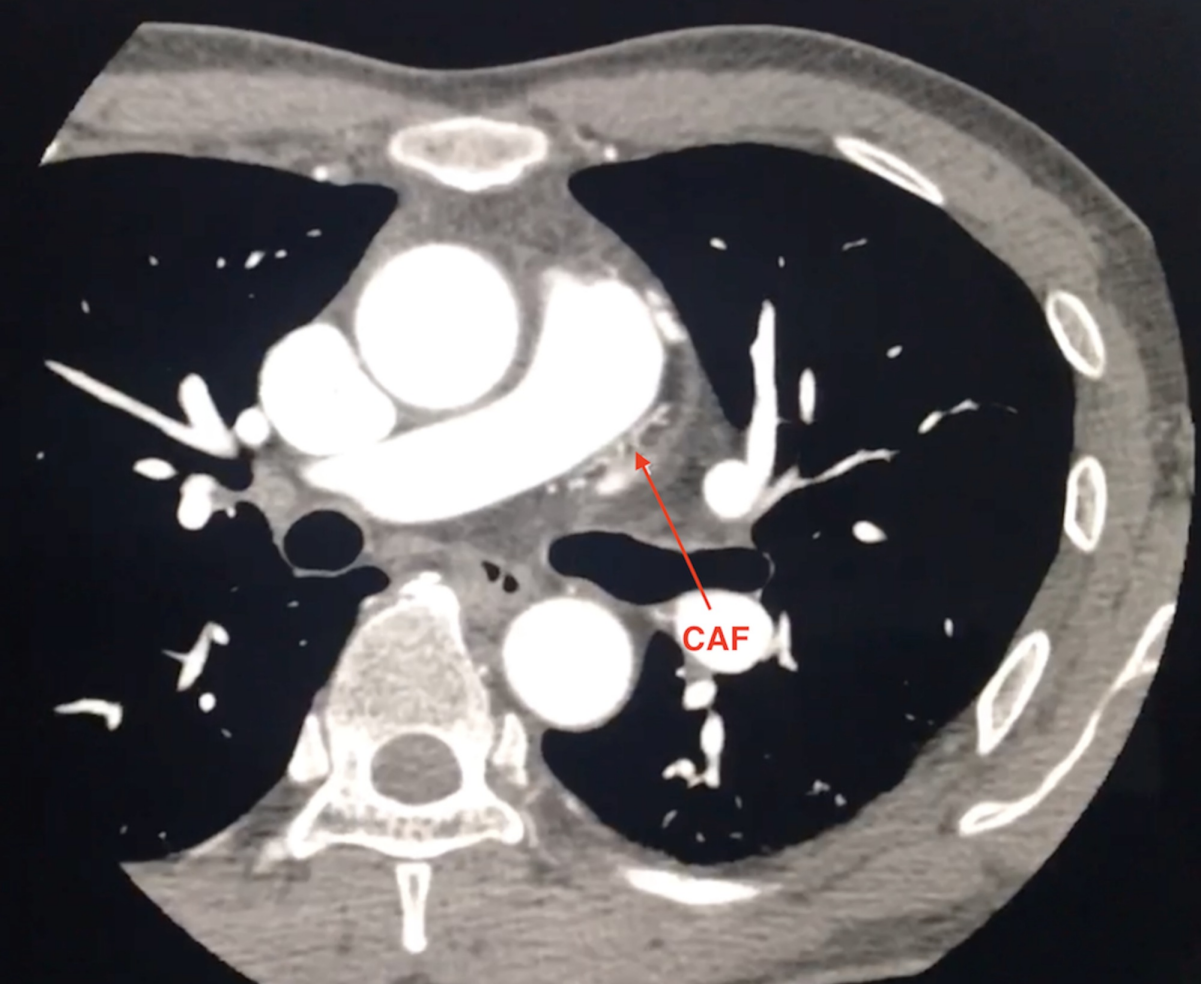
Axial CTA: Network of vessels seen along the PA trunk with loss of intervening fat plane suggestive of a fistulous connection CTA, computed tomography angiography; PA, pulmonary artery

Coronary computed tomography (CT) angiogram was performed showing a tortuous branch distal to the left main coronary bifurcation arising from left anterior descending (LAD) artery and coursing within the epicardial fat along the lateral aspect of the pulmonary outflow tract, as seen in Figures 2-3.

Management

Attempts to coil the fistula via the pulmonary circulation proved unsuccessful due to multiple connections and the large diameter of the fistula. Oximetry data to quantitate the shunt was not performed as the fistula was being terminated into the pulmonary artery. The transcatheter route was discussed with the structural team. The structural team deemed the transcatheter approach unsafe in our patient due to the large bore of the fistula, multiple branches communicating from LAD to the PA, and the close proximity of the fistula to the left-main. Robotic-arm assisted ligation was attempted by cardiothoracic surgery. In the operating room, the robotic arm revealed a 1-cm long feeding vessel coming off the LAD with only one visualized branch corresponding with the result of cardiac catheterization. A network of vessels communicating with the PA was also noted past the feeding branch, which was not isolated during the procedure. The fistula was isolated and ligated close to the LAD. TEE and angiogram were used to confirm the flow to the PA before and after ligation as seen in Video 2.

**Video 2:** CAF isolated and ligated close to LAD CAF, coronary artery fistula; LAD, left anterior descending artery

This resulted in patient recovery without any complications, and he remained asymptomatic.

## Discussion

CAF is a known but rare entity first described in 1865 by Winchester DE [6]. It is an abnormal communication between an epicardial coronary artery and a cardiac chamber or a primary vessel (vena cava, sub-pulmonary veins, pulmonary artery, mediastinal vessels, or coronary sinus) bypassing the capillary bed. CAFs are mostly solitary, but at times there could be multiple micro fistulas [6-7]. Their reported incidence is 0.2% to 1.2%, but the incidence of CAFs is on the rise over the last decade due to the wide use of echocardiography and angiography [6]. CAFs are reported to arise more from the right coronary system than the left, and more than 90% of them drain into the venous circulation [6,8]. Although most of them are congenital, they can present at any age. Acquired causes of CAF have been reported which may occur as a complication of acute MI, angioplasty, CABG, endomyocardial biopsy, or trauma. A rare association of CAF with hypertrophic cardiomyopathy has been reported in the literature and is yet to be delineated [6]. CAF has hemodynamics of an extracardiac left to right shunt when it connects to a right-sided cardiac chamber and resembles aortic insufficiency when connects to a left-sided chamber [8].

The patients with CAF often remain asymptomatic. Whether or not a patient develops symptoms depends on the severity of the left to right shunt and the degree of volume overload produced by the fistula [6]. The complications of CAF include pulmonary hypertension, acute myocardial infarction, sudden cardiac death, coronary steal, congestive heart failure, endocarditis, stroke, arrhythmias, coronary aneurysm, and superior vena cava syndrome [7]. The risk of complications increases with age [9]. Coronary angiography remains the gold standard for diagnosis. It can reliably demonstrate the proximal part of CAF and allows assessment of the size and number of fistulas. Transthoracic echocardiography combined with Doppler color flow imaging, transesophageal echocardiography, magnetic resonance imaging, and contrast-enhanced multidetector tomography can be used as an adjunct to coronary angiography [10].

Spontaneous closure of CAF is uncommon [6]. Treatment for asymptomatic fistulas without significant shunting remains controversial. It has been suggested to decrease future cardiovascular complications, such as infective endocarditis, heart failure or pulmonary hypertension but data are lacking [11]. The usual indications for treatment include symptomatology in combination with the proximal location of the fistula, a single drain site, extra-anatomic termination of the fistula away from normal coronaries, older patient age, and absence of joint cardiac disorders requiring surgical intervention [10]. Trans-catheter techniques are recommended provided there is the absence of multiple fistulas, absence of large branch vessels, single narrow draining site and safe accessibility to the artery supplying the fistula [12]. Transcatheter techniques such as detachable occlusion coils and balloons, amplatzer vascular plugs and umbrella devices, covered stents and histoacryl resins have been previously used to close the CAF [13-14]. Procedural complications such as myocardial infarction, device embolization, fistula dissection, arrhythmias, and death have been previously reported in such cases [14].

The abstract of this article has been presented at the Cardiovascular Research Technologies (CRT) Conference in 2019. "Abstract: Vindhyal M, Boppana VS Non-Atherosclerotic Coronary Angina. Cardiovascular Research Technologies (CRT) Conference, May 2-5 2019."

## Conclusions

Surgical ligation and transcatheter techniques are known treatment options for CAF with similar success rates and mortality and morbidity outcomes. Transcatheter techniques can be used to close CAF provided there is a single narrow draining site that can be safely accessible. Our patient presented with symptoms of stable angina which was unmasked during exercise stress testing. His symptoms are explained by the “steal” phenomenon where coronary blood flow is shunted to the pulmonary artery at the expense of myocardium, resulting in angina. The patient's symptoms abated after successful ligation of the fistula using a robotic arm.
